# Oxy-Inflammatory Profile of Finishers and No-Finishers in an Extreme Ultra-Endurance Trail Race: The 866 km Transpyrénéa

**DOI:** 10.3390/ijms27104295

**Published:** 2026-05-12

**Authors:** Simona Mrakic-Sposta, Maristella Gussoni, Federica Mrakic-Sposta, Guido Giardini, Lorenza Pratali, Michela Montorsi, Alessandro Tonacci, Cinzia Dellanoce, Massimo Martinelli, Alessandra Vezzoli

**Affiliations:** 1 Institute of Clinical Physiology, National Council of Research (IFC-CNR), (Milan-Pisa), Piazza Ospedale Maggiore, 3, 20164 Milan, Italy; maristella.gussoni@unimi.it (M.G.); lorenza.pratali@cnr.it (L.P.); michela.montorsi@uniroma5.it (M.M.); alessandro.tonacci@cnr.it (A.T.); cinziacarla.dellanoce@cnr.it (C.D.); 2 Società Italiana Medicina di Montagna, SIMeM, 35138 Padova, Italy; federica.mrakic_sposta@humanitas.it (F.M.-S.); ggiardini@ausl.vda.it (G.G.); massimo.martinelli@isti.cnr.it (M.M.); 3 IRCCS Humanitas Research Hospital, Via Manzoni 56, Rozzano, 20089 Milan, Italy; 4 Neurology and Neurophysiology Department, Mountain Medicine Center Valle d’Aosta Regional Hospital Umberto Parini, 11100 Aosta, Italy; 5 Institute of Information Science and Technologies “A. Faedo”, National Council of Research (CNR), 56124 Pisa, Italy

**Keywords:** micro-invasive analytic technique, oxidative stress, Electron Paramagnetic Resonance, extreme mountain ultra-marathon

## Abstract

This study investigates the bio-physiological responses occurring under extreme stress conditions and the characterization of the oxy-inflammatory profile of Finishers (FRs) and NoFinishers (NFRs) athletes during the time course and following the Transpyrénéa, an 866 km extreme ultra-race across the French Pyrenees with an altitude difference of 52,900+ m ascent. Thirty-nine experienced ultra-marathon runners (age 43.5 ± 9.1 years; weight 72.1 ± 11.1 kg; BMI 23.3 ± 2.6 kg/m^2^) were studied using minimally invasive methods on capillary blood and urine samples obtained at baseline (T0), during (T1, 2, 3) and at the end (T4) of the race. Reactive Oxygen Species (ROS) production, total antioxidant capacity (TAC), oxidative damage (8-hydroxy-2-deoxy Guanosine: 8-OH-dG and 8-isoprostane: 8-isoPGF2α), inflammatory (IL-6), nitric oxide pathway (NOx and 3-NT), neopterin, and hematologic (lactate, and hematocrit) biomarkers were assessed. In both FR and NFR athletes a marked systemic increase in ROS, oxidative and nitrosative damage, inflammation, transient immune-renal dysfunction and lactate release were detected throughout the race. Compared to FRs, NFRs displayed significant differences concerning ROS production at T0, 8-isoPGF2-α at T0, T1 and T2, and perceived exertion (RPE score) at T2. These data potentially reflect enhanced adaptative responses to training and metabolic efficacy in FRs, allowing them to better tolerate extreme physiological stress.

## 1. Introduction

RandoRaids are ultra-endurance competitions, considered a mix of “Chemin Solidaires”, “Grandes Randonnées” and Non-Stop Raid Nature. Their peculiarity is that they are often conducted in semi-self-sufficiency and run for extremely long distances often in impervious and not marked environments.

Ultra-endurance running is an interesting, unique model to test the human body’s limits. Indeed, significant effects on cognitive and sensory functions [1,2,3,4], physical condition [5], and oxidative stress and inflammation [6,7] in ultra-trailers have already been observed. Those effects are mainly caused by a mix of altitude exposure [8] and environmental conditions [9,10], including cold, heat and hypoxia, as well as muscular fatigue [11], dehydration [12] and sleep deprivation [13]. On the contrary, very few data can be found regarding competitions, misperceptions [14,15] and hallucinatory experiences in ultra-trailer and high-altitude runners [16] as well as lipidomic analysis in ultramarathons in cold environments (Canada-692 km) [17]. However, changes in oxidative stress during ultra-long events such as the TransPyrénéa race (a 866 km-long run) have not previously been investigated. It has long been known that oxidative stress plays a key role in many physiological and pathological states [18,19]. As such, an imbalance between oxidants and antioxidants leads to damage of macromolecules such as proteins, deoxyribonucleic acid (DNA) or lipids as well as antioxidant compounds such as glutathione (GSH) [20]. In fact, it has been shown that exhaustive and prolonged running exercise can produce oxidative stress, inflammation, and transient renal impairment [6,20,21,22,23]. In addition, physical activity can improve circulation through various mechanisms, such as the increase of nitric oxide (NO) production, by affecting vascular endothelial nitric oxide synthase [24]. Furthermore, Radák and colleagues [25] reported that exhaustive aerobic exercise causes an increase in the nitration and carbonylation of serum proteins.

Given these premises, the aim of the present study was to elucidate the impact of the TransPyrénéa race, a RandoRaid 866 Km long running competition, on oxidative stress, the NO pathway, inflammation, and immune and metabolic adaptations under extreme prolonged exertion. To minimize the burden on athletes competing under extreme conditions, all measurements were exclusively performed by adopting minimally invasive approaches, including capillary blood sampling and urine collection.

In fact, the use of minimally invasive sampling techniques is of paramount importance in studies involving endurance athletes and/or extreme environments, where conventional venous blood withdrawal could be impractical or ethically challenging. This methodological approach has been successfully adopted and validated in previous investigations conducted by our research group [26,27,28].

## 2. Results

### 2.1. Anthropometric, Physiological, Hematological Parameters, and Physical Fatigue

Of the 39 athletes initially enrolled (T0), 27 subjects reached Mérens-les-Vals (T1) (after an average of 60.0 ± 21.3 h) and were re-evaluated with respect to T0. Nineteen of them (16 males, mean age 43.1 ± 9.1 years) successfully reached Bagnères-de-Luchon (T2) after 185.9 ± 23.7 h and were re-assessed, while 13 (11 males; mean age 43.8 ± 7.6 years) completed the competition in 363.3 ± 33.1 h (Table 1). At La Pierre St. Martin (T3), 16 athletes reached the *LB* (life base); unfortunately, due to logistic issues, we could test only five of them (four males; 41.8 ± 7.4 years). The body composition and physiological and hematological parameters of the athletes measured throughout the race are reported in Table 1. No significant differences were found between Finishers (FRs) and No-Finishers (NFRs) at T0 for the anthropometric, physiological and hematological parameters. The only significant difference was found in the recent training history (days per week) (see Table 1).

Significant differences were found in the RPE scale in both FRs and NFRs. At the end of the competition (T4) the perceived physical fatigue in FRs was significantly higher compared to T2: 8.84 ± 0.55 vs. 7.66 ± 1.05. In NFR, at T2, the RPE score was higher compared to T1: 9.21 ± 0.81 vs. 7.91 ± 1.12 (Table 1).

### 2.2. ROS Production Rate, Antioxidant Capacity and Oxidative Damage

**Figure 1:**
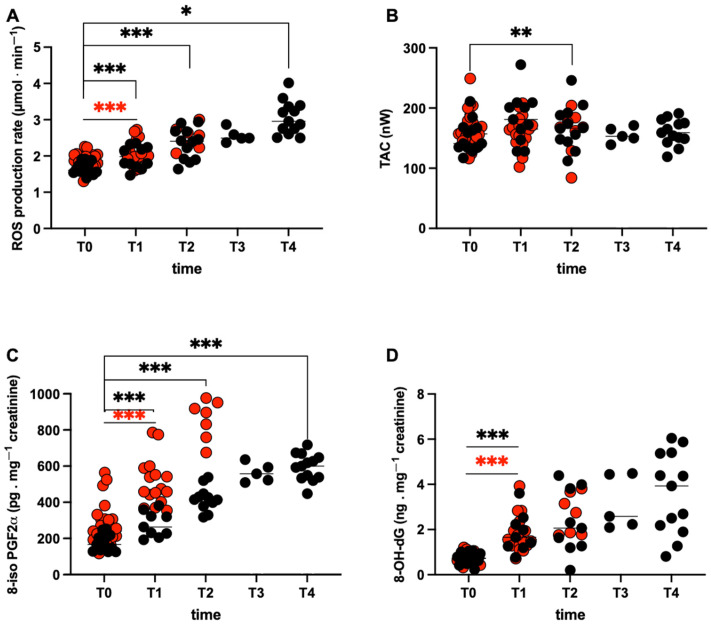
**Race effects on Oxidative Stress.** Data obtained in the two athletes’ groups are shown: FRs (black symbols), NFRs (red symbols). Individual values for: (**A**) ROS production rate (μmol·min^−1^) detected by EPR technique; (**B**) TAC (nW); (**C**) lipid peroxidation (8-iso-PGF2-α pg·mg^−1^ creatinine) and (**D**) DNA oxidation (8-OH-dG ng·mg^−1^ creatinine). Significant changes over time are compared to pre competition (T0) in both groups (symbols * *p* < 0.05; ** *p* < 0.01; and *** *p* < 0.001).

The Transpyrénéa race induced a widespread increase in the ROS production rate in capillary blood. The time course of the ROS production rate levels (mean ± SD) in the two groups of runners (FRs and NFRs) is shown in Figure 1A. Starting from significantly different basal levels (see Figure 4A), the ROS production significantly increased at T1 in both groups: NFRs (T1: 2.05 ± 0.33 vs. T0: 1.84 ± 0.22 μmol·min^−1^; *p* < 0.001) and FRs (T1: 1.94 ± 0.31 vs. T0: 1.66 ± 0.17 μmol·min^−1^; *p* < 0.001). Moreover, in FRs, ROS levels showed a continuous significant increase at T2 (2.33 ± 0.43 μmol·min^−1^; *p* < 0.001) and at the end of the race (T4: 3.06 ± 0.46 μmol·min^−1^; *p* < 0.029) with respect to Pre-Race. On the contrary, as shown in Figure 1B, the antioxidant capacity levels (mM), starting from not significantly different basal values, significantly changed only in FRs at T2 (168.83 ± 36.07; *p* < 0.008).

The kinetics of the oxidative damage biomarkers concentrations displayed in Figure 1C (8-iso PGF2α) and Figure 1D (8-hydroxy-2-deoxy Guanosine) showed a similar difference at T1. No significant differences were found between the levels calculated for FRs and NFRs at Pre-Race in DNA oxidation. As shown in Figure 1C, starting from significantly different basal values, 8-iso PGF2α (Figure 4B) significantly increased at T1 in NFRs (T1: 503.80 ± 130.40 vs. T0: 267.30 ± 109.40 pg·mg^−1^creatinine; *p* < 0.001); while, in FRs, lipids peroxidation levels were found to increase at T1 (278.52 ± 69.50 pg·mg^−1^creatinine; *p* < 0.001), T2 (419.77 ± 67.75 pg·mg^−1^creatinine; *p* < 0.001), and at the end of the race (T4: 595.00 ± 75.00 pg·mg^−1^creatinine; *p* < 0.001). DNA damage data are reported in Figure 1D: 8-OH-dG significantly increased at T1 in both groups: NFRs (T1: 1.80 ± 0.77 vs. T0: 0.77 ± 0.22 ng·mg^−1^creatinine; *p* < 0.001), and FRs (T1: 1.85 ± 0.85 vs. T0: 0.73 ± 0.25 ng·mg^−1^creatinine; *p* < 0.001).

### 2.3. Inflammatory State and Indicators of Metabolic and Immune Activation

**Figure 2:**
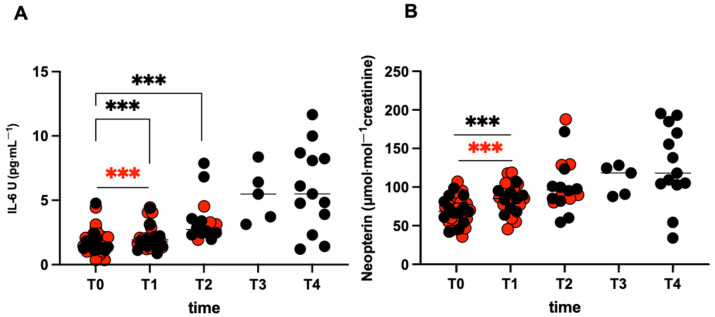
**Race effects on the inflammatory state and immune activation.** Data obtained from the two athletes’ groups are displayed: FRs (black symbols), NFRs (red symbols). Individual values of: (**A**) IL-6U (pg·mL^−1^) and (**B**) neopterin (μmol·mol^−1^creatinine). Significant changes over time are compared to pre competition (T0) in both groups (symbol *** *p* < 0.001).

The measured IL-6U urine levels, displayed in Figure 2A, showed a significant increase in the inflammatory state in both NFRs and FRs. IL-6U was significantly increased at T1 in NFRs (T1: 2.25 ± 1.00 vs. T0: 1.66 ± 0.88 pg·mL^−1^; *p* < 0.002), and at T1 and T2 in FRs (T1: 1.94 ± 0.90, T2: 3.63 ± 2.03 vs. T0: 1.75 ± 0.98 pg·mL^−1^; *p* < 0.001). The time course of neopterin levels (Figure 2B) showed a significant immune activation at T1 in both NFRs (T1: 85.78 ± 20.82 vs. T0: 67.74 ± 17.37 μmol·mol^−1^creatinine; *p* < 0.001), and FRs (T1: 84.24 ± 13.87 vs. T0: 69.90 ± 17.01 μmol·mol^−1^creatinine; *p* < 0.001).

### 2.4. Nitric Oxide

**Figure 3:**
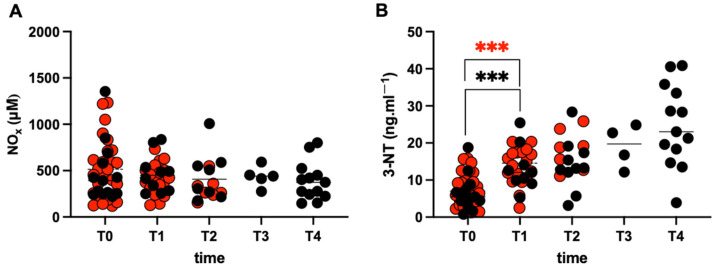
**Race effects on nitric oxide levels**. Data obtained from the two groups of athletes are shown: FRs (black symbols), NFRs (red symbols). Individual values for: (**A**) NOx (mM) and (**B**) 3-NT (ng·mL^−1^). Significant changes over time are compared to pre competition (T0) in both groups (symbol: *** *p* < 0.001).

Figure 3A shows no significant differences in the urinary NOx metabolite (Nitrite (NO_2_−) + Nitrate (NO_3_−)) concentration data (μM) collected on both NFRs and FRs. On the contrary, a significant increase in the 3-nitrotyrosine (3-NT) levels (Figure 3B) was assessed in both groups at T1: NFRs (T1: 13.67 ± 4.89 ng·mL^−1^ vs. T0: 7.58 ± 4.33 ng·mL^−1^; *p* < 0.001), and FRs (T1: 13.24 ± 6.13 vs. T0: 6.53 ± 4.79 ng·mL^−1^; *p* < 0.001). At baseline, no significant differences between NFRs and FRs were found in either markers’ concentrations.

### 2.5. Comparative Analyses Between Groups (FR vs. NFR)

A linear mixed-effects model (LMM) analysis was performed for all biomarkers in order to compare the two different groups.

**Figure 4:**
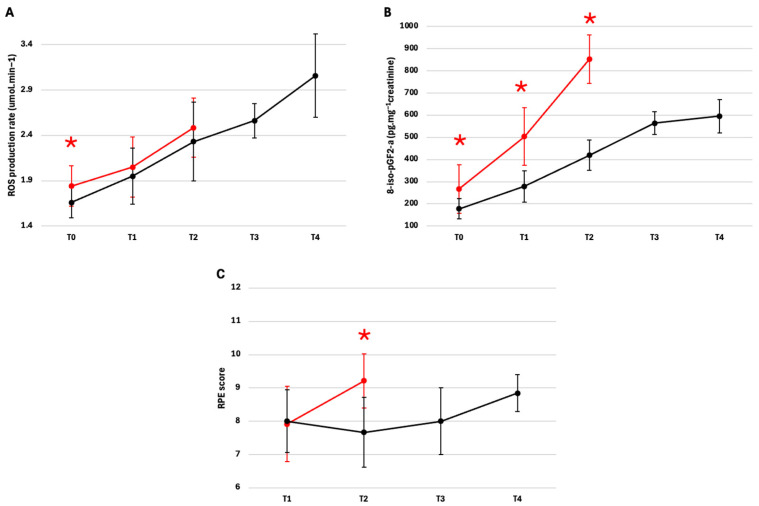
Comparative changes in (**A**) ROS, (**B**) 8-iso-pGF2α, and (**C**) RPE score, in FRs (black line) and NFRs (red line). Data are shown as mean ± 95% CI. ROS and 8-isoprostane were measured from T0 to T4, while BORG from T1 to T4. Significant differences * *p* < 0.05.

A significant difference in ROS production was measured between the two groups at T0, with higher mean values in NFRs with respect to FRs (mean difference = 0.1833; 95% CI: 0.0546–0.3120, *p* = 0.0067; p-FDR = 0.0202; Hedges’ g = 0.8740), as shown in Figure 4A. At T1 and T2, no statistically significant differences emerged, while at T3 and T4, the comparison could not be made due to the absence of NFR subjects.No significant between-group differences were found for neopterin at the analyzed shared time points. The same was observed for IL-6U, despite an increase in mean values over time. In contrast, 8-iso-pGF2α was significantly higher in the NFRs than in the FRs at T0 (mean difference = 89.3640; 95% CI: 39.7358–138.9923, *p* = 0.0039; p-FDR= 0.0039; Hedges’ g = 0.9319). The magnitude of the between-group difference further increased at T1 (mean difference = 225.2410; 95% CI: 142.5180–307.9641, *p* = 0.0000098; p-FDR = 0.000014; Hedges’ g = 1.9279) and T2 (mean difference = 432.4424; 95% CI: 328.0383–536.8465, *p* = 0.0000062; p-FDR = 0.000014; Hedges’ g = 4.8011), as shown in Figure 4B.

At the shared time points, no significant differences between-groups were detected for 8-OH-dG, NOx, and 3-NT. Regarding the RPE score, a significant difference was detected at T2, with higher values in NFRs than in FRs (mean difference = 1.5476; 95% CI: 0.6315–2.4638, *p* = 0.0026; p-FDR = 0.0051; Hedges’ g = 1.5193), as shown in Figure 4C. Detailed analyses are reported in Supplementary Tables S1 and S2.

## 3. Discussion

The Transpyrénéa race represents an extreme model of prolonged physiological stress, due to the overall duration of the event (up to 360 h), the significant elevation gain and the highly variable environmental conditions. The progressive reduction in the number of assessable athletes along the route, as obtained in this study, is consistent with literature data on ultra-endurance competitions of exceptional duration, where withdrawals from the race can exceed 50% even at the early stages of it [6]. Out of the 39 athletes initially enrolled, only 27 reached Mérens-les-Vals (about 69%, in approximately 60 h of racing), and less than half completed the entire event. These data reflect the high physiological and psychological burden imposed by this multi-day competition, in line with previous studies on ultramarathons >200 km, which report progressively increasing dropout rates with increased duration and cumulative elevation gain [29,30]. The enrolled subjects, subsequently stratified into FRs and NFRs for analysis, were homogeneous at T0 (baseline measurements) with respect to age, anthropometric, physiological, and hematological parameters. The only significant difference concerned the recent training history (days/week), with NFRs reporting lower training week exposure compared to FRs (approximately −1.4 days/week). Anthropometric and physiological data demonstrated a significant reduction in body and fat mass, often accompanied by muscle mass loss, attributable to a strongly negative energy balance and an increase in protein catabolism in FR [6,17,31]. Compared to the literature, our data are consistent and even amplified. Indeed, the extremely small number of evaluable athletes at La Pierre St. Martin (n = 5), while representing a methodological limitation, indirectly highlights the logistical and physiological difficulties inherent in field studies of such extreme/extraordinary events. However, in previous studies on extreme ultramarathons, even small samples have been considered informative, given the unique physiological stress induced within this framework [17].

A significant ROS difference was measured between the groups at T0, with higher mean values in the NFRs with respect to FRs. According to our findings, the TransPyrénéa race induced a marked and progressive increase in ROS production in capillary blood, confirming that multi-day ultramarathons represent one of the most powerful physiological stimuli for oxidative stress activation. The significant increase in ROS production levels at T1, measured in both FRs (+17.5) and NFRs (+11%), was consistent with what has been reported in numerous studies on ultra-endurance events, where high oxygen consumption, prolonged mitochondrial activation, and muscle inflammation were found to contribute to free radicals overproduction [6,32,33]. The continued progression of ROS increase in FRs until the race end is noteworthy (T4: +84%). This finding suggests that the duration of exposure to effort represents the main determinant of ROS production in multi-day races. Recent studies indicate that, in extreme duration events (>5–7 days), ROS production can accumulate over time, exceeding the body’s acute adaptive capacities, even in highly trained athletes [6,34]. While EPR provides a direct and quantitative ROS measurements, we acknowledge that no single assay can fully capture the complexity of oxidative stress in vivo, which encompasses multiple reactive species, antioxidant defenses, and cellular compartmentalization. Additionally, blood ROS measurements cannot fully reflect localized oxidative events in specific tissues (e.g., skeletal muscle and/or endothelium). Our results should be interpreted as a robust estimation of systemic ROS dynamics, while recognizing the broader complexity highlighted by Murphy et al. (2022) [35]. Moreover, the physical fatigue scale (RPE score) showed a higher perceived exertion in NFRs compared to FRs. This result could suggest that an elevated effort perception may play a critical role in the decision to discontinue an endurance race. According to the literature, our results are consistent with previous studies indicating that RPE is a key determinant of endurance performance and pacing strategies, and that athletes who can better regulate perceived effort are more likely to successfully complete prolonged events [36,37,38].

In addition to the mechanical stress of running, ultra-endurance events, such as the Transpyrénéa, provide a natural model of intermittent hypoxia due to repeated changes in altitude. Intermittent hypoxia, independent of exercise intensity, can increase ROS production primarily via the xanthine oxidase pathway [39]. In fact, hypoxic exposure leads to ATP degradation and hypoxanthine accumulation, which is subsequently metabolized by xanthine oxidase upon reoxygenation, accompanied by superoxide radical generation, thereby contributing to oxidative stress [40]. The exercise itself can simulate intermittent hypoxia at the tissue level, creating cycles of reduced oxygen availability and reperfusion further amplifying ROS production [41,42,43]. Finally, the combination of mechanical effort and repeated hypoxia–reoxygenation cycles likely synergistically increases oxidative stress in ultra-endurance athletes.

Despite the marked increase in ROS production, total antioxidant capacity (TAC) shows a significant increase in FRs at T2, indicating a more effective buffering response to oxidative stress than NFRs. This is ascribable to the mobilization of reserves (T1 + 20%; T2 + 11%), as previously described by Nikolaidis and colleagues [35]. This result is consistent with the evidence that, under prolonged stress conditions, endogenous antioxidant systems can maintain relatively stable levels; however, these are insufficient to counteract excessive ROS production, leading to the so-called “functional oxidative stress state” [27,34,44]. Such decoupling between ROS and TAC has already been described in long-duration ultramarathons [6,27].

The presence of lipid peroxidation and/or oxidative DNA damage further strengthens this interpretation. An early increase in these markers was detected in both groups just at T1. The significant difference in the 8-isoprostanes production and DNA oxidation between the two groups (Figure 4C,D) could indicate a lower capacity to tolerate the initial oxidative stress in NFRs, potentially contributing to the race failure. In the literature, early elevated levels of isoprostanes and 8-OH-dG have been associated with increased fatigue, muscle damage, and reduced performance in endurance exercise [45,46]. Conversely, FRs experienced a gradual and continuous lipid peroxidation progression, showing significant increases at T2, and T4, suggesting that, despite the presence of physiological and metabolic adaptations that allow for race completion, prolonged stress exposure inevitably induces an accumulation of lipid oxidative damage. Recent studies on multi-day ultramarathons and races over 300 km confirm that oxidative damage can persist and increase until the end of the event, regardless of the training level [47,48,49].

It is well established that long-distance running constitutes an extreme physiological challenge to the whole body and that prolonged aerobic exercise leads to inflammatory markers alterations [47,48,50,51]. Our data show a significant increase in IL-6U just at T1 in both groups (FRs +12%; and NFRs +38%), with a progression of the inflammatory response in FRs at T2. This progression is consistent with what has been reported in long-duration ultra-endurance races, where IL-6 acts as a central mediator of the systemic inflammatory response and as an adaptive metabolic signal in response to prolonged effort [52,53,54,55]. Furthermore, it is known that, during intense and prolonged exercise, IL-6 is also produced by skeletal muscles, and its increase is correlated with the duration and the intensity of the effort, the decreased energy reserves, and the muscle damage [56,57]. The trend observed in FRs, with IL-6U values continuing to increase up to 5.90 ± 3.28 pg mL^−1^ at T4, suggests a cumulative inflammatory burden, associated with the ability to complete the race despite the high systemic stress. Recent studies on ultramarathons of over 200 km report similar increases in IL-6 and other pro-inflammatory cytokines (TNF-α, IL-1β), with peaks reported in the final stages of the competition [58,59].

Regarding urinary neopterin, the significant increase detected in both groups at T1, suggests immune system stimulation. Transient increases in creatinine are often observed, due to muscle metabolism, dehydration, altered renal hemodynamics, and possible transient renal impairment, as also reported in other studies on extreme ultramarathons [6,60]. Similarly, neopterin reflects immune activation and oxidative stress: its levels can rise independently of renal clearance, particularly under conditions of prolonged exercise where inflammation and ROS production become elevated [61]. In fact, neopterin is an indirect marker of macrophage activation and IFN-γ-mediated immune response: elevated values during or after prolonged competitions may reflect oxidative stress and systemic inflammation, as well as possible alterations in renal function due to dehydration, reduced renal perfusion, and prolonged effort [62]. The neopterin increase suggests that immune activation and inflammation are inevitable responses to extreme-duration ultra-endurance races. The similarities between the TransPyrenea race, despite its unique characteristics, with those of the Tor des Géant, validate the idea that similar oxidative stress and inflammatory profiles, and immune responses can be considered as “physiological fingerprints” of ultra-prolonged events [6].

NOx metabolites (NO_2_^−^ + NO_3_^−^), did not significantly change during the race in either NFRs or FRs, although a decreasing trend was observed in NFRs. This result may be explained by multiple mechanisms. Despite the extreme physiological stress imposed by an ultramarathon race, systemic NO production could be regulated, under oxidative conditions, by a possible balance between increased NO synthesis and its rapid consumption. Similar observations have been reported in endurance athletes, where prolonged exercise does not necessarily lead to sustained elevations in NOx levels [6,63,64,65,66]. Conversely, a marked and significant increase in 3-nitrotyrosine (3-NT) at T1, a well-established biomarker of nitrosative stress and protein nitration, was measured in both groups, albeit with different temporal profiles. Compared to baseline (T0), in both groups, 3-NT levels increased significantly at T1 (+80%), indicating an early activation of nitrosative stress pathways. Such a premature increase may reflect an inadequate antioxidant response and an impaired ability to cope with the excessive reactive nitrogen species generated during a prolonged ultra-endurance exercise, which could contribute to fatigue and an inability to complete the race. On the other hand, the increase in 3-nitrotyrosine (3-NT), despite stable NOx levels, reflects enhanced NO scavenging by ROS rather than preserved endothelial NO. NO is a key mediator of the endothelial function but rapidly reacts with superoxide to form peroxynitrite, leading to protein nitration. Stable NOx may thus indicate a balance between production and its rapid consumption. The large 3-NT rise highlights increased peroxynitrite formation and elevated nitrosative stress, suggesting that NO is diverted from physiological targets rather than truly maintained [67,68,69,70].

As previously reported [6,44,45,46,47], ultra endurance exercise markedly stimulates ROS production and oxidative stress, strongly suggesting that exercise duration and intensity are key modulators of ROS generation, consistent with the negative progression of race distance and oxidative responses previously reported by Vezzoli et al. [47]. In line with the present data, the TransPyrénéa competition study showed that NFRs tended to exhibit lower antioxidant capacity and higher oxidative damage biomarkers at mid-race, even though overall ROS production increased in both FRs and NFRs [71].

FRs and NFRs showed distinct physiological and performance profiles. FRs, and ultra endurance athletes in general, often show different effects on oxidative stress compared with NFR, potentially due to training adaptation, antioxidant capacity, and/or metabolic efficiency. In line with this, FRs appear to be characterized by greater endurance performance and more stable pacing strategies throughout the race, suggesting more effective fatigue management. On the contrary, NFRs tended to display early signs of physiological strain and a reduced ability to maintain performance. Indeed, the data of the present study, collected at the same race times from NFRs vs FRs showed different results regarding ROS production rate, inflammation and oxidative and nitrosative damage.

## 4. Materials and Methods

### 4.1. Snapshot of the TransPyrénéa Race

**Figure 5:**
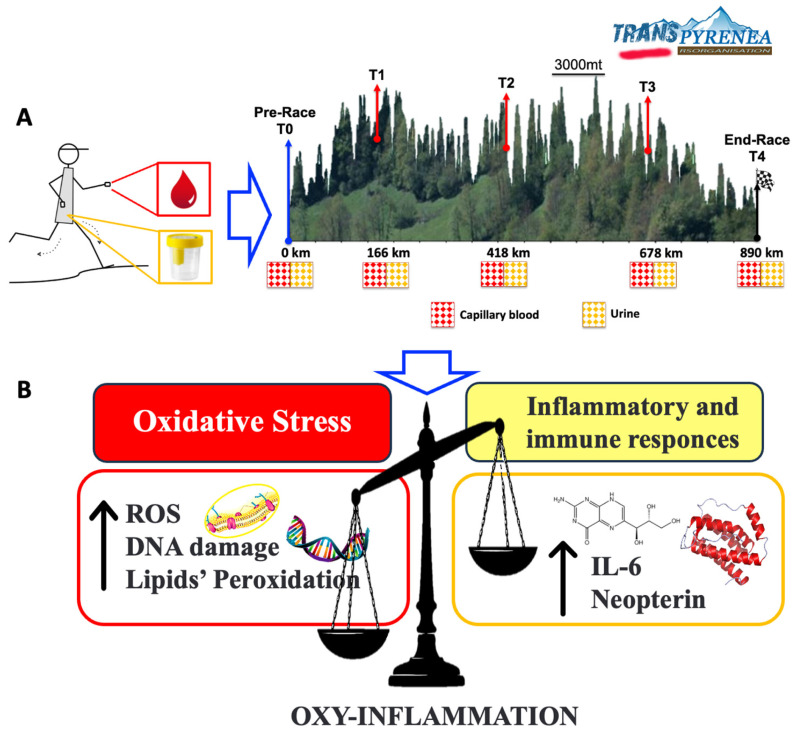
(**A**) TransPyrénéa race elevation profile (Modified by http://www.transpyrenea.fr), and experimental timeline scheme of the protocol adopted to monitor the effects of the ultramarathon competition; (**B**) synopsis of the study findings.

The study was performed during the first edition of the RandoRaid “TransPyrénéa” organized from 19th July to 4th August 2016; a 866 km long run that started near the Mediterranean Sea, in Le Perthus, and ended in Hendaye, on the Atlantic Ocean, following the entire length of the French Pyrenees (The Great Barrier) with an altitude difference of over 52,900+ m ascent. The race runs mainly following the Grande Randonnée path called GR10, passing through 5 French departments, 3 major regions and hundreds of towns and villages and touching peaks between 2.500 m and beyond 3.000 m altitude (Figure 5).

Athletes from 38 countries had to complete the race in semi-self-sufficiency (70% food self-sufficiency), managing the available time in compliance with the strict competition rules. The TransPyrénéa, had 286 starters and 83 finishers (29%). The maximum time allowed for race completion was 400 h (about 16 days) with 22 checkpoints (CPs), and three life bases (LBs, 166, 418, 678 km respectively). The current record (2025) is 11 days 2 h 45 min. The competition was divided into seven stages with thirty-five aid and rest stations where runners could pause to rest or sleep. The organizing committee did not impose any rules regarding the use of these stations, and the winner was determined by who completed the race in the shortest time, deciding independently when and how long to stop for rest or nutrition. Five testing sessions were scheduled for our study. The “Baseline” session (T0) took place in Le Perthus (km 0, altitude 420 m) 1 or 2 days before the race, depending on the availability of the athletes. Participants followed at least 24–48 h of reduced activity/rest [15], prior to the measurements. After the first quarter of the race, a second session was held at the LB in Mérens-les-Vals (T1, km 166, altitude 1060 m), while at halfway a third session was conducted at the LB in Bagnères-de-Luchon (T2, km 418, altitude 640 m). At 678 km, at the LB La Pierre St. Martin (T3, altitude 2200 m) a fourth session was performed. At the end of the race, in Hendaye (T4, km 866, altitude 0 m), athletes were evaluated for the fifth time, within an hour of their arrival and after concluding all physical effort. All measurements were conducted all together at rest and immediately after stopping at the T1, T2, T3 and T4 (end) times (Figure 5A,B).

It is important to highlight that the need to conduct the physiological measurements during the race imposed the creation of mobile and/or on-site laboratory facilities for immediate analysis. To this aim, field-ready laboratory stations were built up, equipped for capillary blood collection and point-of-care assays, allowing the rapid measurement of ROS, lactate, hemoglobin, urinary samples collection, and other physiological parameters directly adjacent to aid stations throughout the competition.

### 4.2. Subjects

Thirty-nine experienced ultra-marathon runners (35 males, 4 female, age (ys) 43.5 ± 9.1; weight (Kg) 72.1 ± 11.1; BMI (Kg/m^2^) 23.3 ± 2.6) were enrolled to voluntarily participate in the study. All subjects were informed of the experimental procedures and signed a written informed consent outlining the study requirements. The procedure was conducted according to the Declaration of Helsinki and approval was obtained from the institutional Ethics Committee of the Aosta Hospital (n. 895; 31 August 2015), Italy. Each participant was provided with a questionnaire to evaluate their health status (e.g., hypertension, hypercholesterolemia, diabetes, and smoking habits), and ultra-endurance experiences was submitted. All athletes enjoyed good health, were non-smokers, and reported about 19.5 ± 15.5 years of ultra-endurance experience. No food-specific diets and/or limitations to the use of vitamin/minerals supplements, herbs and medications were imposed. Anthropometric parameters were determined by bipolar bio-impedentiometry (TBF-300A Body Composition Analyzer; Tanita Corporation, Arlington Heights, IL, USA). Blood pressure (BP) was assessed using a standard cuff sphygmomanometer; peripheral oxygen saturation (SaO_2_) and Heart Rate (HR) (Oximetry-Ohmeda TuffSat-GE Healthcare, Helsinki, Finland) were measured as well (see Table 1).

### 4.3. Capillary Blood and Urine Samples Collection

Capillary blood and urine samples were collected from the athletes. In each session of the race and for each recruited subject, capillary blood (about 500 μL) was taken from the fingertip and ROS production rate, antioxidant capacity, lactate, glucose concentration and hematocrit were immediately determined. Urine samples were also collected during the T0 to T4 sessions, by voluntary voiding into a sterile containers provided to the subjects. Urine samples were stored at −80 °C and thawed only once before analysis, which was performed within one month of collection.

#### 4.3.1. Capillary Blood Measurements

*ROS.* A X-band EPR spectrometer (E-Scan-Bruker BioSpin, GmbH, Billerica, MA USA) was adopted to assess the ROS production rate at the constant temperature of 37 °C, stabilized by a Temperature and Gas Controller “Bio III” unit, interfaced to the instrument. The CMH (1-hydroxy-3-methoxycarbonyl-2,2,5,5-tetramethylpyrrolidine) spin probe was used for ROS trapping in the capillary blood (50 μL). CMH reflects a global ROS burden (superoxide, peroxyl radicals, hydroxyl radicals), while the CP^•^ (3-Carboxy-2,2,5,5-tetramethyl-1-pyrrolidinyloxy) stable radical, used as external reference, returned an absolute quantitative estimation of ROS production. The followed procedure was previously validated and described [6,27,72]. Acquisition parameters: microwave frequency: 9.652 GHz; modulation frequency: 86 kHz; modulation amplitude: 2.28 G; sweep width: 60 G; microwave power: 21.90 mW; number of scans: 10; receiver gain: 3.17·10^1^. All spectra were recorded using same acquisition parameters and processed withthe standard software supplied by Bruker (Win EPR System, V. 2.11).

*Antioxidant capacity.* EDEL potentiostat electrochemical analyser (Edel Therapeutics, Lausanne, Switzerland) equipped with a redox sensor was used to measure the blood reducing capacity on a capillary blood sample (10 μL). This measurement This previously described technique provides an index of the overall antioxidant status [6,27].

*Lactate.* Capillary blood (20 µL) was collected from a fingertip to determine blood lactate concentration ([La]_b_) by using an enzymatic method (Biosen 5030; EKF, Eppendorf Italia, Milano, Italy) [6].

*Hematocrit (Hct).* Changes (%) in plasma volume were assessed from capillary blood samples by centrifuging heparinized blood in micro-hematocrit capillaries (Hirschmann Laborgeräte GmbH and Co.; Eberstadt, Germany) at 10.000 rpm for 5 min (4223 Centrifuge ALC, Sacem S.r.l.; Cremona, Italy) and directly analyzed. Blood samples were measured in duplicate [6].

#### 4.3.2. Urine Measurements

*Lipid peroxidation and DNA damage*. 8-isoprostane (8-iso-PGF2 α) and 8-OH-2-deoxyguanosine (8-OH-dG) concentrations, were assessed in urine by using commercially available assay EIA kits (Cayman Chemical, Ann Arbor, MI, USA, Item No. 516351 and Item No. 89320 respectively). These biomarkers provide quantitative indices of oxidative damage to lipids and DNA, respectively, reflecting systemic oxidative stress levels. The analyses were carried out in accordance with the manufacturer’s instructions [26,28]. The sample concentrations were determined using standard curves. Samples and standards were spectrophotometrically read at a wavelength of 412 nm. The coefficient of variation (CV) indicated by the manufacturer was as follows: inter- CV of 11.5% and 8.4%; intra-assay CV of 8.9% and 6.2% for 8-iso-PGF2 α and 8-OH-dG respectivelty.

*Inflammatory marker*. Urinary Interleukin 6 (IL-6-U) levels were determined by a ELISA kit (Cayman Chemical, Ann Arbor, MI, USA, Item No. 501030), according to the manufacturer’s instructions, as previously reported [6,26,73]. Samples were spectrophotometrically read at a wavelength of 450 nm; CV indicated by the manufacturer, inter-assay range: 5.40–26.22%; intra-assay range: 4.12–9.28%.

*Nitrate/Nitrite levels and Nitrotyrosine.* Urine Nitrate/Nitrite (NOx) levels determination was performed by spectrophotometric method using Griess reagent, utilizing a commercial colorimetric assay kit (Cayman Chemical, Ann Arbor, MI, USA, cat N° 780001), at 545 nm. A linear calibration curve was previously obtained from pure nitrite and nitrate standards [74]. For nitrotyrosine (3-NT) determination, an assay kit (cat no EU2560; FineTest, Wuhan, China) was used, by adopting a competitive-ELISA method. The 3-NT urine concentration was spectrophotometrically determined at a wavelength of 450 nm. The previously described analysis was carried out in accordance with the manufacturer’s instructions [26]; CV indicated by the manufacturer: inter-assay: 3.4%, and 5.68%; intra-assay: 2.7% and 6.42%, respectivelty for NOx and 3-NT.

All the spectrophotometric determinations were carried out in duplicate by a microplate reader spectrophotometer (Infinite M200, Tecan Group Ltd., Männedorf, Switzerland).

*Indicators of Metabolic and Immune Activation.* Urinary creatinine and neopterin concentrations were measured by the high-pressure liquid chromatography (HPLC) method as previously described [6,75]. The calibration curves were found linear over the 0.125–1 μmol·L^−1^ range level for neopterin, and 1.25–10 mmol·L^−1^ for creatinine. The inter-assay and intra-assay CV were <5%.

Each urinary marker was standardized based on the amount of excreted creatinine, since a 24 h urine collection was not possible. While in healthy human subjects in the absence of renal disease, the excretion rate of creatinine remains relatively constant [76].

### 4.4. Scale for Assessment of Physical Fatigue

Perceived exertion was evaluated immediately upon the arrival in every LB (from T1 to T4). Scores were based on physical sensations and muscle fatigue, assessed using the Borg Rate of Perceived Exertion scale (RPE) [36].

### 4.5. Statistical Analysis

All statistical analyses were performed using JASP, an open-source statistical software package for macOS (JASP 0.19.3, Amsterdam, The Netherlands) and Python (3.14.5) on Linux, using statsmodels library. Figures were generated using the GraphPad Prism package for Mac (GraphPad Prism 10.6.1, Software Inc. San Diego, CA, USA). A linear mixed-effects model (LMM) was employed given the longitudinal nature of the study design, the presence of repeated measurements within the same subjects over time (intragroup), and the occurrence of missing data due to partecipant dropout during the race. For each biomarker, ROS, TAC, neopterin, IL-6U, 8-isoprostane, 8-OH-dG, NOx, 3-NT, and RPE scores, a comparison was performed between the NFRs and FRs at the different sampling times. A correction for multiple comparisons was applied using the Benjamini–Hochberg False Discovery Rate (FDR). Furthermore, to jointly evaluate the time effect, the FR status, and their interaction, the model could only be applied at the times common to both groups. The dataset structure showed an asymmetry in the distribution of the longitudinal data, since the times actually shared between the two groups were mainly T0, T1, and T2, while NFRs were not represented at the subsequent times, T3 and T4, limiting the possibility of estimating those models. Hedges’ g was calculated to quantify the effect size between the two groups. Statistical significance was set at *p* < 0.05. Data are here presented as means ± SD. Additionally, the change Δ% is reported as Δ% = ((post competition—pre competition)/pre competition) × 100. An a priori power analysis was performed to estimate the required sample size using the G*Power software (G*Power 3.1.9, http://www.psycho.uni-duesseldorf.de/abteilungen/aap/gpower3/, accessed on 19 May 2016). Assuming a statistical power of 80%, and a significant alpha level α = 0.05, the analysis indicated that a minimum of 11 subjects was required to detect a significant effect, with the ROS production rate chosen as the primary outcome. The final sample size of recruited runners included in the study exceeded the minimun requried number of partecipants.

## 5. Conclusions

In conclusion, our results confirm that the TransPyrénéa competition induces significant anthropometric, physiological, oxidative stress, and inflammatory changes that are progressive along the route and dependent on the duration of exposure to effort. These data appear consistent with the existing literature on ultra-endurance competitions but are amplified by the exceptional duration of the race, making the TransPyrénéa a unique model for studying the limits of human adaptation to prolonged effort. Moreover, it represents a unique physiological model for studying the limits of human redox homeostasis under conditions of extreme and prolonged stress. These findings may have practical relevance for athletes, coaches, and other stakeholders involved in ultra endurance events. By providing a detailed profile of oxidative and nitrosative stress during extreme prolonged exercise, the data can help practitioners monitor physiological strain and optimize training and recovery strategies. Knowledge of systemic ROS and related biomarkers of oxidative damage and inflammation dynamics could also inform nutrition and supplementation approaches aimed at mitigating the oxy-inflammation income. Furthermore, federations and race organizers might use these data to enhance supporting infrastructures, such as the positioning aid stations and/or medical monitoring, at critical stages of ultra endurance competitions. Overall, our results offer insights surely that contribute to athlete health, performance optimization, and evidence-based event planning in the context of extreme endurance sports.

## Figures and Tables

**Table 1:** Mean (±SD) of the parameters collected from the athletes taken altogether at Pre-Race or referred to the FR and NFR groups at different racing times (from T0 to T4). Training experiences; scale for the asssessment of physical fatigue: RPE: Rating of Perceived Exertion score; body composition analysis: Wgt: weight; BMI: Body Mass Index; FM: Fat Mass; TBW: Total Body Water. Physiological data: SaO_2_: arterial oxygen saturation; HR: Heart Rate; SBP: Systolic Blood Pressure; DBP: Diastolic Blood Pressure; haematolgical values: Hct:Hematocrit, and [La]b(mM): Blood Lactate. Significant differences compared to Pre-Race: * *p* < 0.05. In the RPE scale, significant differences in FRs compared to T2 # *p* < 0.05; and in NFR scompared to T1 ## *p* < 0.05.

	**All Athetes**	**FINISHERS (FRs)** ***n* = 13 (11 M)**					**NO-FINISHERS** **(NFRs) *n* = 27**		
	**T0** ***n* = 39** **(34 males)**	**T0** **420 m**	**T1** **1060 m**	**T2** **640 m**	**T3** **2200 m**	**T4** **sea level**	**T0** **(*n* = 27)** **420 m**	**T1** **(*n* = 18)** **1060 m**	**T2** **(*n* = 7)** **640 m**
**Age** (years)	43.5 ± 9.1	43.8 ± 7.5	-	-	-	-	43.9 ± 9.5	-	-
**Training experiences**									
**Recent training history** (d/week)	5.3 ± 1.9	6.2 ± 1.9	-	-	-	-	4.8 ± 1.8 *	-	-
**Recent training’s history** (h/week)	11.5 ± 6.6	13.3 ± 6.8	-	-	-	-	10.5 ± 6.5	-	-
**Training** **history** (year)	19.5 ± 15.5	15.6 ± 13.3	-	-	-	-	21.3 ± 16.3	-	-
**Time of walking in race** (h)	-	-	61.1 ± 7.6	124.2 ± 13.5	185.9 ± 23.7	361.5 ± 33.8	-	58.8 ± 25.7	112.7 ± 26.4
**Scale for Physical Fatigue assessment**									
**RPE** (score)	-	-	8.0 ± 0.94	7.66 ± 1.05	8.0 ± 1.0	8.84 ± 0.55 #	-	7.91 ± 1.12	9.21 ± 0.81 ##
**Body composition analysis**									
	**T0**	**T0**	**T1**	**T2**	**T3**	**T4**	**T0**	**T1**	**T2**
**Wgt** (kg) **BMI** (kg/m^2^) **FM** (kg) **TBW** (kg)	72.1 ± 11.1 23.3 ± 2.6 5.40 ± 3.51 49.1 ± 7.5	71.7 ± 9.5 23.2 ± 1.9 4.70 ± 2.5 49.0 ± 7.4	70.4 ± 5.4 22.1 ± 1.4 3.88 ± 2.09 49.6 ± 4.0	72.2 ± 6.9 22.3 ± 1.4 * 2.16 ± 1.32 * 50.2 ± 4.6	67.8 ± 7.1 21.4 ± 1.6 * 2.92 ± 1.21 * 47.1 ± 6.4 *	67.3 ± 9.9 21.1 ± 1.4 * 2.34 ± 0.96 * 44.8 ± 6.5 *	73.0 ± 11.9 23.4 ± 2.8 5.40 ± 2.46 49.4 ± 7.4	69.6 ± 11.3 22.5 ± 2.9 4.30 ± 3.90 48.2 ± 7.5	68.3 ± 10.4 23.2 ± 2.0 - -
**Physiological data**									
		**FINISHERS (FRs)**					**NO**-**FINISHERS** **(NFRs)**		
	**T0**	**T0**	**T1**	**T2**	**T3**	**T4**	**T0**	**T1**	**T2**
**SaO_2_** (%) **HR** (BPM) **SBP** (mmHg) **DBP** (mmHg)	97.1 ± 1.3 64.3 ± 10.1 128.3 ± 20.9 78.5 ± 14.9	97.2 ± 0.9 64.8 ± 10.1 126.3 ± 20.9 78.5 ± 14.8	96.2 ± 1.1 71.2 ± 11.9 124.2 ± 18.8 77.1 ± 15.7	96.7 ± 0.9 63.1 ± 6.1 128.3 ± 20.9 78.5 ± 14.9	97.2 ± 1.3 59.8 ± 12.1 130.1 ± 14.4 70.2 ± 12.3	97.2 ± 1.3 68.5 ± 12.4 126.3 ± 12.6 68.7 ± 7.2 *	97.0 ± 1.5 65.9 ± 9.6 139.7 ± 22.7 82.5 ± 10.7	95.4 ± 1.2 * 74.2 ± 12.8 129.5 ± 11 * 69.5 ± 10.1 *	97.6 ± 1.9 65.6 ± 7.47 120.0 ± 8.1 * 65.5 ± 3.4 *
**Hematological values**									
	**T0**	**T0**	**T1**	**T2**	**T3**	**T4**	**T0**	**T1**	**T2**
**Lactate** [La]b(mM) **Hct** (%)	1.1 ± 0.9 44.6 ± 2.6	1.1 ± 0.8 44.6 ± 2.5	2.9 ± 2.5 * 42.4 ± 2.1	3.2 ± 1.9 * 41.2 ± 3.3	4.1 ± 3.9 * 39.7 ± 3.4 *	4.4 ± 3.5 * 41.2 ± 3.4	1.1 ± 0.9 44.1 ± 2.8	3.1 ± 2.8 * 42.6 ± 3.1	3.5 ± 2.4 * 40.3 ± 2.5

## Data Availability

The original contributions presented in this study are included in the article/Supplementary Materials. Further inquiries can be directed to the corresponding authors.

## Supplementary Materials

The following supporting information can be downloaded at: https://www.mdpi.com/article/10.3390/ijms27104295/s1.

## Abbreviations

The following abbreviations are used in this manuscript:

BMI	Body Mass Index
DBP	Diastolic Blood Pressure
EPR	Electron Paramagnetic Resonance
FM	Fat Mass
FRs	Finishers
Hct	Haematocrit
HPLC	High-Performance Liquid Chromatography
HR	Heart Rate
IL-6	Interleukin-6
Lac	Lactate
NFRs	No-Finishers
NOx	Nitric Oxide metabolites
OxS	Oxidative Stress
RPE	Rating of Perceived Exertion
SBP	Systolic Blood Pressure
TAC	Total Antioxidant Capacity
TBW	Total Body Water
3-NT	3-nitrotyrosine
8-iso-PGF2α	8-isoprostane
8-OH-dG	8-hydroxy-2-deoxy Guanosine
