# Supplementary material for: Oxy-Inflammatory Profile of Finishers and No-Finishers in an Extreme Ultra-Endurance Trail Race: The 866 km Transpyrénéa
Source: Int J Mol Sci. 2026 May 12;27(10):4295. doi: 10.3390/ijms27104295 (PMC13207344; doi:10.3390/ijms27104295)
Supplement: Supplementary file 1 [file ijms-27-04295-s001.zip › ijms-4204028-supplementary.pdf]

Unified comparison table: ROS, RPE score, and 8-iso-pGF2-a

Biomarker	Time	n NFR/ FR	NFR Mean±SD	FR Mean±SD	Test	Mean difference F0-F1 (95% CI)	p	p-FDR	Hedges' g
ROS	T0	27/13	1.8416±0.2208	1.6583±0.1681	Welch t-test	0.1833 (0.0546 to 0.3120)	0.0067	0.0202	0.874
ROS	T1	18/11	2.0499±0.3310	1.9485±0.3088	Mann-Whitney U	0.1014 (-0.1503 to 0.3530)	0.544	0.544	0.3051
ROS	T2	7/12	2.4836±0.3255	2.3313±0.4348	Welch t-test	0.1524 (-0.2208 to 0.5255)	0.399	0.544	0.3642
ROS	T3	0/5	-	2.5617±0.1882	Not testable	-			
ROS	T4	0/13	-	3.0582±0.4578	Not testable	-			
BORG	T1	18/10	7.9167±1.1279	8.0000±0.9428	Mann-Whitney U	'-0.0833 (-0.9123 to 0.7457)	0.8219	0.8219	-0.0758
BORG	T2	7/12	9.2143±0.8092	7.6667±1.0517	Welch t-test	1.5476 (0.6315 to 2.4638)	0.0026	0.0051	1.5193
BORG	T3	0/5	-	8.0000±1.0000	Not testable	-			
BORG	T4	0/13	-	8.8462±0.5547	Not testable	-			
8-iso	T0	27/13	267.2851±109.4060	177.9211±45.1969	Mann-Whitney U	89.3640 (39.7358 to 138.9923)	0.0039	0.0039	0.9319
8-iso	T1	16/9	503.7688±130.3696	278.5278±69.5017	Welch t-test	225.2410 (142.5180 to 307.9641)	0.00000987	0.0000148	1.9279
8-iso	T2	7/11	852.2197±109.4133	419.7773±67.7580	Welch t-test	432.4424 (328.0383 to 536.8465)	0.00000627	0.0000148	4.8011
8-iso	T3	0/5	-	564.2080±52.0917	Not testable	-			
8-iso	T4	0/13	-	595.3956±74.9955	Not testable	-			

Table 1. Comparisons between group no-Finishers (NFR) and Finishers (FR) at each time point (from T0 to T4) for ROS, RPE scale, and 8-iso-pGF2-α. Value reported as sample size, mean ± standard deviation (SD), statistical test used, mean difference with 95% confidence interval, p-value, false discovery rate-adjusted p-value (FDR), and Hedges' g. Comparisons at T3 and T4 were not testable because no subjects were available in the NFR.

LINEAR MIXED MODEL – time categorial

Mixed Linear Model Regression Results

**Model:**	**MixedLM**	**Dependent Variable:**	**ROS**
No. Observations:	54	Method:	REML
No. Groups:	13	Scale:	0.0939
Min. group size:	2	Log-Likelihood:	-22.2488
Max. group size:	5	Converged:	Yes
Mean group size:	4.2		

	Coef.	Std.Err.	z	P>|z|	[0.025	0.975]
Intercept	1.658	0.097	17.049	0.000	1.468	1.849
C(time, Treatment(reference='T0'))[T.T1]	0.291	0.126	2.299	0.022	0.043	0.539
C(time, Treatment(reference='T0'))[T.T2]	0.664	0.123	5.383	0.000	0.422	0.905
C(time, Treatment(reference='T0'))[T.T3]	0.925	0.166	5.558	0.000	0.599	1.252
C(time, Treatment(reference='T0'))[T.T4]	1.400	0.120	11.644	0.000	1.164	1.635
Group Var	0.029	0.080				

Table 2. Using a linear mixed-effects model with time as a categorical factor, the longitudinal analysis showed a significant increase in ROS over time compared to baseline supporting a progressive increase in ROS, accounting for within-subject dependence through the inclusion of a random intercept.
